# Univariate and Multivariate Analysis of Phosphorus Element in Fertilizers Using Laser-Induced Breakdown Spectroscopy

**DOI:** 10.3390/s19071727

**Published:** 2019-04-11

**Authors:** Baohua Zhang, Pengpeng Ling, Wen Sha, Yongcheng Jiang, Zhifeng Cui

**Affiliations:** 1 School of Electronics and Information Engineering, Anhui University, Hefei 230061, China; 2 School of Electric Engineering and Automation, Anhui University, Hefei 230601, China; ppling@yeah.net (P.L.); ahu001@163.com (W.S.); ycjiang@126.com (Y.J.); 3 Institute of Atomic and Molecular Physics, Anhui Normal University, Wuhu 241000, China; zfcui@mail.ahnu.edu.cn

**Keywords:** fertilizer, phosphorus element, laser-induced breakdown spectroscopy, chemometrics

## Abstract

Rapid detection of phosphorus (P) element is beneficial to the control of compound fertilizer production process and is of great significance in the fertilizer industry. The aim of this work was to compare the univariate and multivariate analysis of phosphorus element in compound fertilizers and obtain a reliable and accurate method for rapid detection of phosphorus element. A total of 47 fertilizer samples were collected from the production line; 36 samples were used as a calibration set, and 11 samples were used as a prediction set. The univariate calibration curve was constructed by the intensity of characteristic line and the concentration of P. The linear correlation coefficient was 0.854 as the existence of the matrix effect. In order to eliminate the matrix effect, the internal standardization as the appropriate methodology was used to increase the accuracy. Using silicon (Si) element as an internal element, a linear correlation coefficient of 0.932 was obtained. Furthermore, the chemometrics model of partial least-squares regression (PLSR) was used to analysis the concentration of P in fertilizer. The correlation coefficient was 0.977 and 0.976 for the calibration set and prediction set, respectively. The results indicated that the LIBS technique coupled with PLSR could be a reliable and accurate method in the quantitative determination of P element in complex matrices like compound fertilizers.

## 1. Introduction

The use of compound fertilizers in agriculture to improve soil quality is very common. China’s compound fertilizer use ranks first in the world according to statistics. Fertilization in some areas is extremely unreasonable, causing serious environmental pollution [1]. Phosphorus(P) element is a major nutrient element for crops and is very important in agriculture. Quality control is very important for compound fertilizer manufacturers, and can help guarantee the quality of products. At present, the real-time sensing rapid detection of compound fertilizer production has been mainly manual sampling, sample preparation and laboratory testing. The traditional sensing method of P in compound fertilizers is the phosphomolybdate quinoline gravimetric method [2], which is mature and has high accuracy. However, this sensing detection technique commonly requires the dissolution of the solid sample, which involves the use of high temperatures and strong oxidants. With the development of sensing analysis technology, optical detection methods are increasingly used for the detection of compound fertilizer components, such as flame atomic absorption spectrometry (FAAS), and inductively coupled plasma-mass spectrometry (ICP-MS) [3,4], but, because of a typically high concentration in compound fertilizer, the sensing measurement of P needs to be diluted several times. The aforementioned sensing detection methods are time-consuming, labor intensive, and expensive. As a consequence, the use of the traditional detection techniques increases the systematic errors besides producing large volumes of chemical residues [5].

Laser-induced breakdown spectroscopy (LIBS) is an emerging analytical technique in the current spectroscopic field. The LIBS technique has many advantages, such as in situ detection, real-time, remote sensing capability, multi-elemental analysis, minimal sample preparation, and direct analysis of any state of matter [6]. It has been successfully used in chemical and biological testing [7], water pollution [8], coal combustion [9], agriculture [10], artifacts and jewelry identification [11], space exploration [12], etc. Some works have used the LIBS technique for the analysis of the component of compound fertilizer. Farooq et al. determined P, Mg, and Mn in the fertilizer using LIBS [13]. Quantitative LIBS analysis of phosphorus in 26 different organic and inorganic fertilizers has been reported by Bruno S. Marangoni et al., however, the absolute error of the measurement for the two verification samples is close to 5% [14]. The elements of Cu, K, Mg, Mn, Zn, As, Cd, Cr and Pb in liquid fertilizers were analyzed with LIBS technique by Daniel Fernandes Andrade et al. [15]. S.C. Yao et al. detected phosphorus and potassium elements in the compound fertilizer using LIBS, and the PLS quantitative analysis model was established by using Unscrambler software [16]. Daniel Fandrade et al. have reported an application of LIBS for quantification of the metal elements in solid compound fertilizers [17]. However, quantitative aspects have generally been considered a shortcoming of LIBS, which greatly limits its application. Thus, there are still many problems to be solved prior to routine practical applications.

The univariate calibration considers the emission intensities of excited element and its concentration. However, the fertilizer was a complex sample, which contains many elements of Fe, Si, Mg, Al, and O. All of these elements may produce matrix effects, and also, due to the fluctuations observed in LIBS technique associated with the instruments and sample non-uniformity, many strategies are used for the calibration methods, such as different spectral preprocessing and multivariate calibration models. Internal standardization is a common method used to minimize fluctuations in LIBS technique, which consists of normalizing the analytical signal by an internal signal. Usually, the internal element concentration must be nearly constant [18]. However, the internal element concentration may slightly change from sample to sample, thus, the accuracy of quantitative analysis results still needs to be improved. In chemometrics, partial least-squares regression (PLSR) is one of the multivariate analytical techniques. It is very crucial to reduce the matrix effect when dealing with complex sample [19].

In this work, 47 fertilizer samples provided by the compound fertilizer production line of Hefei Hongsifang Chemical Fertilizer Plant, Anhui, China were used as the testing samples. The concentration of phosphorus in compound fertilizerswas determined by inductively coupled plasma (ICP). The univariate calibration was established by the LIBS intensity and the concentration of P. Then, the internal standardization method and PLSR were used to quantitatively analyze the phosphorus concentration. The main goal of this research is to prove that LIBS technique can be used for on-line rapid detection of phosphorus element in compound fertilizer.

## 2. Materials and Methods

### 2.1. Sample Preparation

In this study, 47 compound fertilizer samples were collected and placed into sealed plastic bags so as to avoid contamination by manufacturer. Since these samples had been pelletized, these samples were smashed by using a grinder and sieved through a 60-mesh screen. 2 g powders from each of 47 fertilizer samples was weighed. All fertilizer powders were pressed into tablets with 25 mm diameter and 5 mm in thickness, using 5 MPa pressure for 1 min (769YP-40C, KQ, Tianjin, China). The actual concentration of P in these samples were analyzed by inductively coupled plasma (ICP). The statistics of the P concentrations in compound fertilizer samples was listed in Table 1.

### 2.2. Experimental Setup

**Figure 1:**
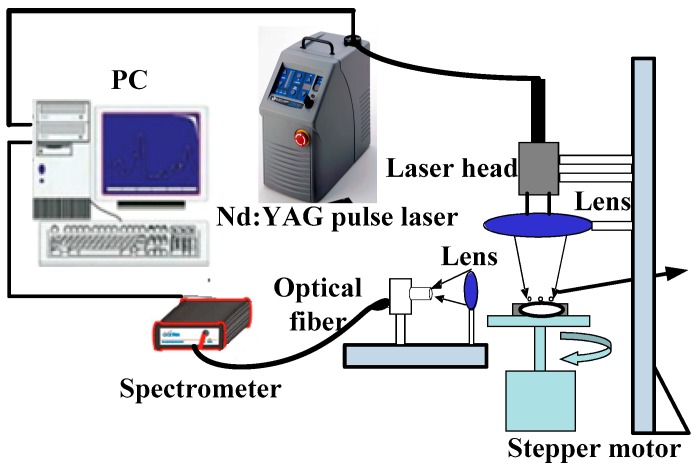
Schematic of LIBS experimental system.

Figure 1 gave out the spectral acquisition system used in this experiment. The laser pulses were generated by using a Q-switched Nd: YAG laser (ICE450, 1064 nm, 6 ns pulse duration, Big Sky Laser Technologies, Morgan Hill, CA, USA; Note that the company has changed its name to Quantel Laser). The laser pulse energy was 100 mJ and focused onto the sample through a lens of focal length 5 cm. The spot size of beam was approximately 0.5 mm and the peak power density on the surface of the compound fertilizer sample reached 2.2 GW/cm^2^.When the plasma generated from the fertilizer sample, a quartz lens with 3.5 cm focal length, which was connected to a four-channel spectrometer (Avantes-ULS2048-USB2, Avantes, Apeldoorn, The Netherlands) via a 200-μm diameter optical fiber, was used to collect the spectra from the plasma. The spectrograph signal was integrated with a charge-coupled device detector. This spectrometer can simultaneously take all spectra in the wavelength ranges of 190–510 and 690–890 nm, and the resolution of the spectrometer was approximately 0.1 nm. The laser Q-switch output was used to trigger the spectrometer, and the spectrometer has a digital delay generator, which can control the gate delay. Here, the Q-switched delay time selected for spectra acquisition was 1.28 μs and the integration time was 1.05ms (spectrometer minimum integration time) [20]. A rotary platform on which the fertilizer sample was placed was rotated uniformly to avoid continuous ablation of the same spot. During the experiment, each sample was measured eight times, and each spectrum collected was an average of 20 laser spot, and during each measurement, the fertilizer sample rotated once by adjusting the speed of the stepper motor.

### 2.3. Chemometrics Methods

In LIBS technique, the calibration curve method, also called univariate analysis, is a traditional quantitative analysis method. The element characteristic line intensity is proportional to the concentration in the sample, when there is no self-absorption [21]. Therefore, the calibration curve can be established by the element concentration and the intensity of LIBS signal. Then, the concentration of an unknown sample can be calculated according to the calibration curve. However, due to the matrix effect, this method is not suitable for quantitative analysis of complex sample. Internal standardization is usually used for quantitative analysis in LIBS technique, which can improve the accuracy of LIBS technique and reduce the fluctuations observed in LIBS measurements, but, there are some principles for selecting the internal standard element [22]. The concentration of the internal standard element should be approximately constant, and the wavelength of the internal standard element should be close to the analytical element. In addition, the excitation potential should be similar for the internal standard element and the analytical element. The coefficient of determination and the root mean squared error, for the calibration set and validation set, were adopted to evaluate the performance of the internal standardization model.

PLSR is widely used for quantitative analysis of LIBS spectra in recent years [23,24]. This method performs quantitative spectral analysis by selecting latent variables [25,26]. Therefore, it is very important to select the latent variables, which directly determined the predictive performance of the calibration model. The PLSR model was established by the LIBS signal intensity and the concentration of P for fertilizer samples. In order to avoid the overfitting of the PLSR model, and also to obtain a reliable and robust PLSR model, full cross-validation was applied. The number of latent variables was determined when the mean squared error was minimum. Furthermore, the statistic parameters for evaluating the performance of PLSR model include the determination coefficient for calibration (R_C_^2^) and prediction (R_P_^2^), the root mean square error for calibration (RMSEC) and prediction (RMSEP), and residual predictive deviation (RPD) [27]. All data processing procedures were compiled with MATLAB.

## 3. Results

### 3.1. Spectral Analysis

**Figure 2:**
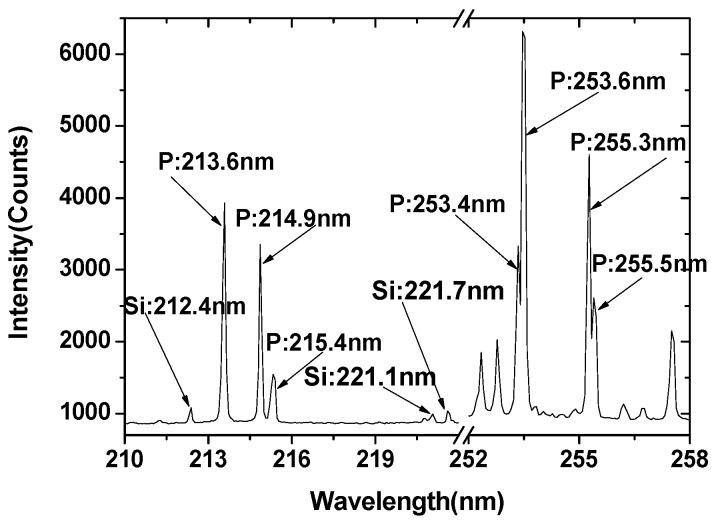
LIBS spectrum of compound fertilizer sample in the ranges of 210–222 and 252–258 nm (n = 20).

The LIBS spectrum of the compound fertilizer pellet (No.1 sample) in the ranges of 210–220 and 250–260 nm is shown in Figure 2, which includes the emission lines of silicon (Si) and P. Compound fertilizer production enterprises generally use phosphate ore as raw material. Thus, Si is one of the main ingredients of compound fertilizers, and the characteristic lines of Si are 212.4 nm, 221.1 nm, and 221.7 nm according to the National Institute of Standards and Technology (NIST) database. It can be seen from Figure 2 that the compound fertilizer sample contains abundant characteristic lines of P element. The feature spectral lines of P element detected by LIBS were 213.6 nm, 214.9 nm, 215.4 nm, 253.4 nm, 253.6 nm, 255.3 nm, and 255.5 nm. The characteristic lines of 253.4 nm and 253.6 nm are interfered by the characteristic line of Fe element in the compound fertilizer. The adjacent peaks of the two characteristic lines of 255.3 nm and 255.5 nm can be clearly distinguished, and Lorentz double peak fitting is needed when fitting the line intensity. The characteristic lines of 213.6 nm, 214.9 nm and 215.4 nm are not disrupted by other elements.

**Figure 3:**
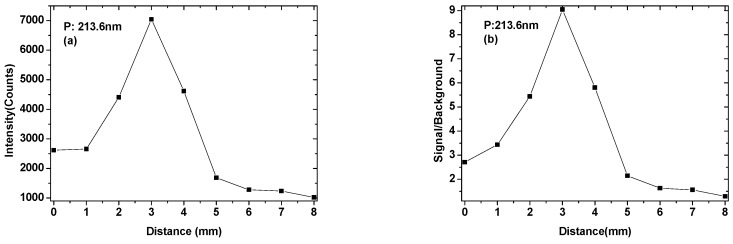
The line intensity of P 213.6 nm (**a**) and signal to background ratio (**b**) as a function of detection distance.

In order to obtain a stable signal, the focus of laser beam was adjusted at the position of the fertilizer sample. When the laser focus located on the surface of the fertilizer sample, the distance was recorded as d = 0 mm. Then, the laser focusing system was adjusted, each time moving the laser focus to the surface of the fertilizer sample 1 mm, up to 8mm. The P: 213.6 nm was selected as the analytical line, the relationship of the line intensity and the signal-to-background ratio (SBR) with the laser focus position was shown in Figure 3. The maximum value of the line intensity and the SBR of P were all located at 3 mm below the surface of the fertilizer sample. When the focus of the laser beam gradually moved downward from the surface of the fertilizer sample, the laser pulse energy was more absorbed by the sample, so that the ablation amount was gradually increased, more atoms and ions were in an excited state. But, as the distance between the laser pulse focus and the surface of the fertilizer sample further increased, the radiant power of the laser pulse on the surface of the fertilizer sample gradually decreased, so that the ablation amount of the composite fertilizer sample decreased. However, when the focus of the laser pulse moved down to a certain distance, it was basically difficult to break down the fertilizer sample, so the line intensity and the SBR of the phosphorus element tended to be stable.

### 3.2. Univariate Analysis

**Figure 4:**
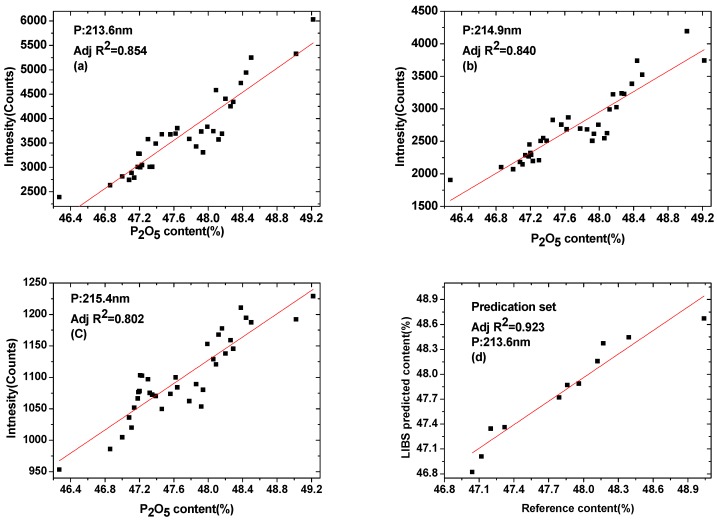
Calibration curves of P spectral line: (**a**) P: 213.6 nm, (**b**) P: 214.9 nm, (**c**) P: 215.4 nm, and (**d**) the relation of LIBS predicted value and reference value for the prediction set.

Before modeling, 47 fertilizer samples were split into a calibration set (36 samples) and a prediction set (11 samples) based on the K-S method. The univariate calibration model was constructed by the line intensity (the height of Lorentz fits) of P versus the corresponding concentration [28]. The content of P is in the range of 46.27–49.22%. Figure 4a–c show the calibration curve of three characteristic lines of P. The spectral line (P: 213.6 nm) that obtained the best modeling result was applied for subsequent analysis. Figure 4a indicates the linear trend between line intensity and concentration with a coefficient of correlationof 0.854. For the prediction set, 11 fertilizer samples were used to estimate the prediction accuracy of the LIBS technology. The predicted content of samples can be obtained by taking the line intensity into the calibration fitting curve. The relation between the reference content and LIBS-predicted content for P is shown in Figure 4d, with an R^2^ value of 0.923. Although the relative error of the prediction set is not very large, but the correlation coefficient should be improved for further quantitative analysis.

### 3.3. Multivariate Analysis

**Figure 5:**
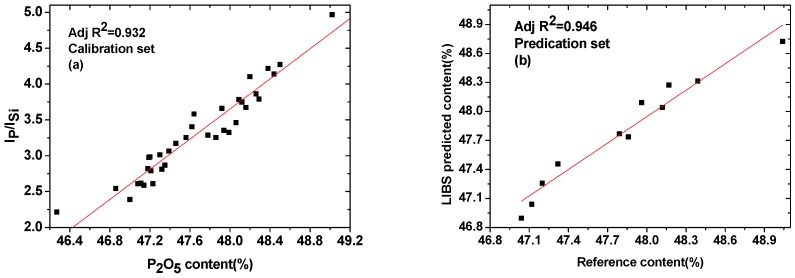
Internal standard method: (**a**) calibration curve using Si as an internal standardelement and (**b**) comparison of P content predicted by LIBS and the referencevalue (ICP).

According the principles for selecting the internal standard element, the characteristic line of Si 212.4 nm was selected as the internal standard line. The internal standard curve was constructed by calculating the lineintensity ratio of the analytical element and the internal standard element. The ratio of P line intensity (213.6 nm) to that of Si (212.4 nm) versus the concentration of P in fertilizers was used for calibration. Figure 5a shows the internal standard curve for P element. The value of R^2^ was obviously improved to 0.932 from 0.854. Similar to the univariate calibration method, the sample content of prediction set were calculated. Figure 5b shows the relation between the reference concentration and LIBS predictedconcentration for P, with R^2^ changing from 0.923 to 0.946. The range of relative error was 0.04–0.65%, which was improved with the univariate calibration method. These results indicate that the internal standard method can partly eliminate the instability of the LIBS signal, but the detection sensitivity and the prediction accuracy still need to be improved.

**Figure 6:**
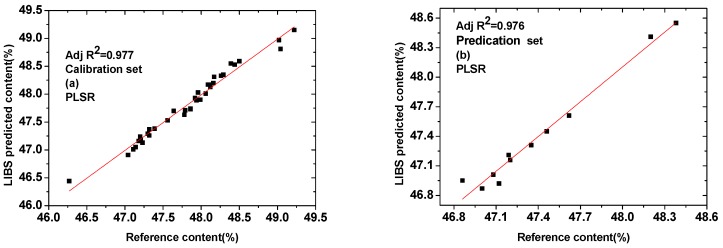
Comparison between LIBS predicted value (PLSR model) and reference value presented in (**a**) thirty-six calibration samples and (**b**) eleven prediction samples.

Because of the complex fertilizer matrix, the analysis focusing only on one line intensity of an element might result in the loss of valid information, which cannot meet the requirements of the quantitative analysis of LIBS. PLSR is one of the multivariate analytical techniques, which can make full use of the spectral information, reduce the matrix effect and improve the accuracy of quantitative analysis. Sample sets were the same as those of the above univariate model and internal standard model. Calibration set was used to construct a model correlating the LIBS signal and the concentration of P; this correlation can later be used to predict concentrations of prediction set. In order to improve the processing speed and avoid overfitting of the model, a proper spectral range was selected for modeling analysis. A reduced spectral range from the full spectrum, which included most of the strong lines of P, was taken into account for PLSR model. The reduced wavelength ranges from 210 to 260 nm for P was used to obtain calibration model. The MATLAB software was used for PLSR. Seven principal components are used to construct the PLSR model. Figure 6a–b shows the calibration and prediction results of PLSR model for P, respectively.

It can be seen from Figure 6 that most of the calibration and prediction data points were distributed around the fitting curve, which indicating that the PLSR model performed well in predicting of P content. The major statistic parameters that determine capacity of the regression model are R_C_^2^, R_P_^2^, RMSEC, RMSEP and RPD. All of the parameters for both calibration and prediction sets are presented in Table 2. The determination coefficient for calibration set (R_C_^2^) was changed from 0.932 to 0.977, while for prediction (R_P_^2^) set was improved to 0.976 from 0.946. The slope for both the calibration and prediction sets was close to one, which showed a strong correlation between predicted and reference values. Meanwhile, the value of RMSEC and RMSEP were 0.117 and 0.113, respectively, which was better than the results reported by S.C. Yao et al. [16]. In [16], the RMSEC was 0.234, and in this case, RPD value exceeded 5, which suggesting that the established prediction models can be employed for robust quantitative analysis. In real agricultural applications, the measured value of P content in compound fertilizer is 1.5% plus or minus the standard value, and the absolute difference between different laboratory measurements is not more than 0.5% (ISO 5315: 1984, MOD). In this paper, the difference between the predicted value of LIBS and the reference value isin the range of 0.02%–0.32%, which can fit the requirement. The total relative error for calibration and prediction set was 5.83% and 2.08%, respectively, which was better than the results reported by Bruno S. Marangoni et al. [14]. In [14], the average error of 15% found in cross-validation of LIBS quantification appeared feasible for P quantification in fertilizers. It is demonstrated that PLSR model can be developed to predict the concentrations of unknown samples. Thus, the measurement accuracy of this result can meet the measurement requirements [29].

## 4. Discussion

In this paper, the LIBS technique was used for univariate and multivariate analyses of P element in compound fertilizers. Forty-seven samples were provided by Hongsifang production. The calibration curve was established based on the three selected emission lines and the concentration of P. The results showed that the characteristic line of 213.6 nm was most suitable for establishing calibration curves. Due to the occurrence of matrix effects, the prediction accuracy of the method could not be achieved by applying the univariate calibration method, which only using I_P_ as the variable. The internal standardization method based on Si was naturally present in the samples, which showed the proper correction of the P signal. The internal standard method was found to be better than the calibration method because the correlation coefficient for the calibration set was changed from 0.854 to 0.932. Moreover, the range of relative errors are 0.04–0.65%. Thus, the internal standard method can improve the accuracy of the measurements in some extent. Then, PLSR was used as a multivariate analytical technique for analysis of compound fertilizers in pellet form. From the results of PLSR regression, calibration and prediction models were obtained for P element with very good correlation coefficients. The values of RMSEC and RMSEP were 0.117 and 0.113, with RPD value of 5.31. All of these results demonstrated that the PLSR regression method can improve the accuracy of LIBS measurement, and the results in this study can provide the basis of real-time analysis of P in compound fertilizer.

## Figures and Tables

**Table 1:** Statistics of the Effective Constituents of Compound Fertilizer Samples.

Properties	P_2_O_5_ (%)
Minimum value	46.27
Maximum value	49.22
Mean value	47.731
Standard deviation values	0.617

**Table 2:** R^2^ Regression Coefficients, RMSEC, RMSEP, RPD for Calibration and Prediction Curve.

Parameters	R_C_^2^	RMSEC	R_P_^2^	RMSEP	RPD
Values	0.977	0.117	0.976	0.113	5.31
